# A Phase of Modern Medicine

**Published:** 1889-10-12

**Authors:** 


### A Phase of Modern Medicine!

It has been cynically observed that if we wish to discover centres of all the moral virtues we must investigate the tombstones of the churchyards, in which the forefathers and foremothers of the present degenerate generation "lie mouldering in their graves." It has also been said that one has nothing to do to make an enemy but to render a person a service; an act which will convert the greatest friend into the bitterest foe. Happily, however, there are no rules without the proverbial exceptions. Medical men, in common with others who "pass their useful lives away" in the service of their fellow-creatures, often complain, or at least feel, if they do not openly complain, that they rarely secure the gratitude of their patients. "He did it for his dirty fee" but too frequently represents the feelings of patients when they get well towards their doctors. Most people when they are ill are saints; when they recover they are less saintly. But we have discovered a royal road by which doctors may not only secure the gratitude of their patients, but also put more money in their purses. We hasten to impart the secret to the profession. It is this: let all turn mountebanks and quack doctors! According to the veracious advertisements of the fraternity, they invariably secure the gratitude of their patients, and, as the sequel will show, fill their pockets with coin.

We have been led into the above reflections by visiting (when *en route* from the meeting of the British Medical Association at Leeds) a certain county town, which shall be nameless. Hearing accidentally a conversation concerning a certain "doctor" who had performed many wonderful cures, and who lectured every day at a named spot in this favoured city, we determined to hear, mark, and inwardly digest. Arriving on the ground, we found the place adorned with gigantic placards announcing the miraculous cures performed by the medicine-man. We had the curiosity to measure one of these placards, and found it to be of canvas, and about two yards by ten. We had also the curiosity to copy the print, which is as follows: "Called back from the grave," in very large letters. "Dear Sir,—I have suffered severely from dropsy for over six months, for two months of which I was confined to my bed. I was enormously swollen, being over sixty inches round the waist, and twenty inches round the leg. I passed hardly any water. I was treated by four doctors, but got no relief. Four weeks ago I began using your remedies—prairie flower and Indian oil. They gave me instant relief, and in a few days I was able to get up, and in a fortnight I was able to go out of doors. I have steadily amended, and am now quite well, and feel as well as ever I did in my life. My doctor had given me up. Thanking you with gratitude for my recovery, I am, gratefully yours, Mrs. Shepherd, wife of Mr. Shepherd, 19, Ferry-hill-terrace, Aberdeen." Various other more or less gigantic placards were to similar effect, and the following, among other names, were used: John D. Jones, Methodist minister, Belfast, cured of lumbago; Rev. T. Burton, St. Andrews, Aberdeen, personal knowledge of people cured; W. Pope, inspector of police, cured; Rev. R. Cornwall, vicar, Emanuel Church, Bristol, bears testimony; Rev. J. Dewar, Rutherford Free Church, Aberdeen, bears testimony; Rev. J. P. St. Clair, Aberdeen, bears testimony; Rev. J. Dann, Townsend Vicarage, Bristol, hearsay evidence; Rev. S. Smith, St. Mayard's, Aberdeen, says "of those I have seen there is not one who has not derived benefit." Now, we do not know whether the gentlemen mentioned above have authorised their names to be used as described; but whether or not the fact remains that the names are so used, and in such a manner that those who run may read. We have simply given some of the names exposed to the public gaze, and doubtless the reader will be struck, as we were, by the number of clergy on the list. Now, clergymen being well educated, and possessing a defined and certain standing, are doubtless sought after to testify to cures by patent medicines, etc. And some clergymen, unfortunately, appear too fond of quackery. Perhaps this arises from an archbishop being permitted for so long to dub a man M.D. Perhaps from the fact that the clergy are so often consulted by their poorer parishioners regarding ailments of the body. Perhaps from an idea that clergymen are *ex officio* qualified to follow the example of their, and our, Lord and Master, and so heal the body as well as the soul. Perhaps from that innate love of prescribing physic so characteristic of the true Briton. However this may be, the fact remains that many clergy are too fond of quackery, and not unfrequently like to have a finger in the doctor's village pie.

But while making these reflections the "doctor" we came to see is arriving. First, we hear a band of brass, if not of music. Then appears on the scene an enormous gilded "machine," drawn by four gaily-decorated horses. On the front part of the machine are seated eight musicians, the back part being, as yet, vacant. For the "doctor" who is to perform therein, comes behind in a handsome carriage and pair. Alighting from this, he mounts the gilded "machine," and announces his intention of drawing teeth gratuitously for any of the numerous company, at least a thousand, assembled. He has not long to wait, for the machine is quickly invaded by men, women, and children desirous of having their aching teeth pulled out. In the space of half an hour the doctor must have "drawn" fifty or sixty teeth, much to the amusement of the multitude of lookers-on, who evidently greatly enjoyed the grimaces made by the sufferers. As a rule, the teeth were extracted with some skill; but now and then flying pieces of broken crowns showed the operator was not infallible. No questions were asked, the person ascending the stage pointed out the tooth, the forceps or other instrument was introduced, and the tooth twisted out, if necessary, by sheer force. And so doubtless many teeth were extracted which the dentist's skill would have saved. Now, some time ago we saw much the same performance taking place at Nice; but the Frenchman pursued a somewhat different system. He did not draw all the teeth presented to his notice, for some, he said, had a worm in them, which he would extract. With an instrument he scooped out the pulp of the tooth, which he handed round as the worm. This may be commended as a hint to the English "doctor."

Having extracted a great many teeth, but not all presenting, our "doctor" now began a speech to the people. He announced that he knew nothing about anatomy or physiology, or chemistry, or anything else that medical men trouble themselves with; but that he had discovered when among the Indians (and, by the by, it should be said the doctor was clothed in leathern garments, supposed to represent an Indian's dress) a remedy for most of the ills that flesh is heir to. After mentioning Jenner, and Watt, and half-a-dozen others who had made discoveries not well received at first, he pathetically observed that such was his case at first, but that now he had conquered. He then asked if there was anyone present affected with rheumatism. At least a dozen so suffering were present, some brought in Bath chairs. At last a rather big man was pushed and hauled on to the machine, his name and address were asked, enquiry was made from the bystanders if any of them knew him, the history of his complaint was detailed—sciatica of the left leg, also affecting the muscles—and then the doctor mentioned that it would be necessary to strip the patient, also inviting several persons of the company to ascend the "machine" and to assist in holding a large buffalo robe round the patient and the doctor. The writer ascended, aided in holding the buffalo robe, and therefore had a full view of the procedure. The lower garments of the sufferer being taken off, the "doctor" commenced rubbing in his specific oil. For a full half hour the "doctor" laboured manfully, perspiration streaming from him. He rubbed, and kneaded, and punched, and rubbed again, using oil copiously, until he must have been quite exhausted. All this time the band was playing, and at least a thousand persons waiting patiently. At length the sufferer's lower garments were adjusted, the buffalo robe removed, and the patient told to stand up and walk. The patient, however, seemed to have some doubt on the subject, but the order was peremptorily repeated, and, to the astonishment of the whole assembly, the man who was carried up one side of the "machine" walked down the ladder on the other side of the "machine," round the "machine," and up again into it, where he was a second time covered with the buffalo robe. Then the "doctor" introduced six persons who had been similarly cured. Names and addresses were given; people were asked if they were acquainted with them; they were caused to describe their former ailments; and then they were made to illustrate their present condition by jumping in the presence of the audience. One man stated he had been afflicted fifteen years, could scarcely walk; had passed recently nine months in the local infirmary, and had "never received 'no benefit' until he used the 'doctor's oil.'" He began to use it last Thursday, and was now "as well as ever he was in his life," and his gratitude was unbounded. "Ah!" said a woman standing next to the writer, "that's like my aunt. Her couldn't lift her hand no how, until her used them oils, and now hers as well as ever her was in her life." After this, the doctor began to sell his wares at 2s. a bottle. Evidently, the oil is simply soap liniment, with, perhaps, a little turpentine in it. What the prairie flower is this deponent sayeth not; but it was described as good for indigestion, constipation, rheumatism, dyspepsia, and, in fact, any ailment afflicting humanity. And the "doctor" reaped a bountiful harvest. He said he sometimes sold ten pounds' worth after one of his exhibitions, and on this occasion must have taken more than such a sum. He then drove away with band playing, and banners waving, doubtless to pastures new, for the day was not half spent.

Now, the query at once arises, were the *cures* displayed *bonâ fide*, or were the persons impostors in collusion with the doctor? The latter idea may be dismissed, for some of the persons "cured" had resided in the locality many years, and were well known to various persons present. The conclusion, therefore, is inevitable that the "cures" were real. But are they permanent or only temporary? Doubtless the latter. And two principal factors are in operation. First, the vigorous friction to which the affected limbs are subjected. Secondly, imagination and its development, faith. It is believed that massage is a substitute, at least in some degree, for exercise, causing as it does increased combustion of tissue. By vigorous rubbing, both nerves and bloodvessels, large and small, are stimulated, causing increased flow of blood through the parts, the disruption and washing away of sharp stagnating crystals of lithic acid, the consequent relief of pain and stiffness, and ability to use the limb. As regards imagination, having, as previously mentioned, just returned from the meeting of the British Medical Association at Leeds, Sir James Crichton Browne's admirable address could not but recur to mind. As a matter of fact, said Sir James, imagination and "faith has an overpowering influence on individuals and nations. Professors of faith-healing, like empirics of all sorts, have wrought modern miracles on hypochondriacal men who have fallen into drivelling egotism; on hysterical girls who have entertained demons not unawares; and on a few sincere, but sensitive persons who can pass at a touch out of one allotropic state of the nervous system into another. . . But let faith-healers commence with skin diseases, woo the *acarus* from its burrow without the aid of brimstone, arrest lupus, or wipe out a nævus, and we shall be ready to follow them with some hope in their campaign against hidden maladies." But at the same time Sir J. Crichton Browne admits that imagination and faith are among the most successful of those psychical agencies by which we may modify the conditions of disease, and he asserts that the medical practitioner "is never more a medical psychologist than when he is practising what is called moral treatment, and is by his manner, demeanour, feeling, and well-chosen words driving out fear, calming turbulent feeling, and encouraging self-control. His success or failure as a practitioner will indeed often depend as much on his expertness in this moral treatment as in his manual dexterity or skill in simples." We have a further illustration of the effects of imagination and faith afforded us by Dr. C. L. Tuckey in his recent book on "Hypnotism," and in his recently-published "cases treated by hypnotism and suggestion." Like the mesmerist of former days, he puts his patient to sleep, and then suggests, or rather orders them, to be well when they awake. And in persons of a certain temperament, and without organic disease, this is often followed by success, of at least a temporary nature. Our "doctor," the subject of this article, pursues much the same plan, substituting massage and oils for sleep; he then commands his patients to walk, and their limbs having been temporarily relieved of peccant material, many of them obey the command. Moreover, the class of people worked upon are chiefly of the lower and uneducated stratum of society. They see immense placards with apparently good names attached as vouchers; they are impressed by the gilded machine, the four horses, the braying band, the Indian dress, and the confident demeanour of the "doctor." Emissaries are going about in public-houses, and wherever people congregate, leading the conversation to the miraculous cures performed, which certainly do not lose in wonderment by repetition. And when people go to the place of meeting to hear the "doctor," they form a favourable opinion of his dexterity from his questionable skill in pulling out teeth.

Now persons are likely to ask why the undoubted temporary cures performed should not be permanent? The answer is sufficiently easy. In the first place, the stimulation afforded to a rheumatic limb can only be of a temporary nature. To produce any effect it must be, as described, very vigorous, and the more vigorous it is the sooner will the nerves, capillaries, and other bloodvessels cease to respond. By over stimulation, and consequent increased activity, the parts lose their sensitiveness, and, in simple language, become tired out. Morbid products are no longer washed away by increased flow of blood, and the last state of the limb becomes worse than the first. Again, the "doctor" will not always be present with his placards, brilliant equipages, strange dress, and confident demeanour, to work on the imagination. And so the sufferers temporarily benefited lapse into their former condition. In the meantime the doctor will be far away, reaping a rich harvest elsewhere. It is passing strange that within a comparatively short distance of Leeds, where some of the most eminent members of the medical profession were congregated, and where science in its highest acceptation was the order of the time, there should be a flagrant exhibition of charlatanism; an exhibition, indeed, which secured the sympathies, admiration, and even enthusiasm of the populace. Let us, take a lesson even from a mountebank. Most persons suffering from a painful malady, such as rheumatism, would assert that even a temporary cure is "better than nothing at all." That relief, even if of short duration, is preferable to continued pain. Do we, therefore, in our hospitals endeavour sufficiently to apply scientifically the means used by this emperic—viz., systematic massage and imagination? Most people are inclined to think we still trust too much to drugs.

